# Pulmonary Arterial Hypertension and Endothelial Dysfunction Is Linked to NADPH Oxidase-Derived Superoxide Formation in Venous Thrombosis and Pulmonary Embolism in Mice

**DOI:** 10.1155/2018/1860513

**Published:** 2018-06-10

**Authors:** Moritz Brandt, Eleni Giokoglu, Venkata Garlapati, Madgalena L. Bochenek, Michael Molitor, Lukas Hobohm, Tanja Schönfelder, Thomas Münzel, Sabine Kossmann, Susanne H. Karbach, Katrin Schäfer, Philip Wenzel

**Affiliations:** ^1^Center for Cardiology – Cardiology I, University Medical Center Mainz, Langenbeckstrasse 1, 55131 Mainz, Germany; ^2^Center for Thrombosis and Hemostasis Mainz, University Medical Center Mainz, Langenbeckstrasse 1, 55131 Mainz, Germany; ^3^Deutsches Zentrum für Herzkreislaufforschung (DZHK) – Partner site Rhine-Main, University Medical Center Mainz, Langenbeckstrasse 1, 55131 Mainz, Germany

## Abstract

Pulmonary embolism (PE) results from deep vein thrombosis (DVT) and can lead to chronic thromboembolic pulmonary hypertension (CTEPH) involving vascular dysfunction. Mechanisms are incompletely understood, in part due to lack of mouse models. We induced PE in C57BL/6 mice by intravenous injection of thrombin (166 U/kg BW), confirmed by a sudden bradycardia, bradypnea, and an increase in pulmonary artery (PA) pressure observed by high-frequency ultrasound. While symptoms resolved rapidly after single thrombin application, repeated PEs resulted in sustained PA-pressure increase, increased PA superoxide formation assessed by oxidative fluorescent microtopography, increased PA gp91^phox^ expression, and endothelial dysfunction assessed by isometric tension studies of isolated PA segments after 24 hours. DVT was modeled in C57BL/6 mice by ligation of the inferior vena cava (IVC). Importantly, small pulmonary emboli could be detected along with a mild phenotype of PA endothelial dysfunction and oxidative stress in the absence of PA-pressure elevation. mRNA expression of plasminogen activator inhibitor-1 was increased in PAs of mice with recurrent PE after repetitive thrombin injections and to a lesser extent in DVT mice. In summary, our data suggest that PA endothelial dysfunction, induced by gp91^phox^-derived ROS, is an early event upon repetitive PE. This phenomenon might help to elucidate the mechanisms of PA dysfunction in the pathogenesis of CTEPH.

## 1. Introduction

Pulmonary arterial hypertension (PAH), defined as a mean pulmonary arterial pressure (PAP) of 25 mmHg or more, is a hemodynamic and pathophysiological state that can be found in multiple clinical conditions. Chronic thromboembolic pulmonary hypertension (CTEPH) is a major cause of pulmonary hypertension when it is not secondary to heart disease or chronic lung disease. It is believed that early in the disease, thrombotic material, mostly originating from large and deep veins in the lower extremities (deep vein thrombosis (DVT)), embolises into the pulmonary vasculature causing acute pulmonary embolism (PE). Acute PE mechanically obstructs pulmonary arteries and reduces the total diameter of the pulmonary vascular bed. The amount of blood that has to pass through the pulmonary vasculature is tied to the oxygen demand of the body and is therefore fixed to a certain range. In order for the same amount of blood to pass the decreased pulmonary vascular bed, a higher perfusion pressure is required and the PAP rises. While this elevation in PAP accompanies almost every PE exceeding a hemodynamically relevant size, PAP elevation is usually transient, as most patients are able to resolve the emboli with only minimal residual abnormalities. However, in up to 9.1% of patients with acute PEs and in over 10% of patients with recurrent PEs, the emboli are not completely resolved and persist in the major pulmonary arteries [1]. A yet unidentified mechanism leads to the persistence of initial pulmonary emboli. A process of ongoing remodeling with fibrosis and hyperplasia of arterial media and intima is initiated, which leads to the development of additional vascular stenoses and occlusions, eventually resulting in the disease termed CTEPH. This condition is characterized by secondary small-vessel arteriopathy of nonoccluded pulmonary arteries, which indicates a mechanism of self-perpetuation in the disease [2]: reduction of vascular diameter by initial thromboembolism(s) with subsequent elevation of PAP and pressure-induced remodeling leading to further reduction of vessel diameter. Patients with untreated CTEPH develop a progressive disease and have a high risk of death from right heart failure. Until today, the mechanisms causing incomplete resolution of pulmonary thromboemboli, underlying the development of fibrotic occlusions together with vascular remodeling of pulmonary resistance vessels, remain unclear. This lack of understanding is in large part due to the paucity of a reliable animal model that reflects the pathophysiology of acute PE and/or CTEPH. In particular, mouse models of acute PE or CTEPH, which would allow the use of genetically modified animals to perform mechanistic in vivo studies, are not broadly available.

We therefore aimed to investigate the effect of single or repetitive sublethal experimental pulmonary embolism on the pulmonary vasculature and pulmonary endothelial dysfunction, further comparing it to the inferior vena cava-stenosis model, a widely used model to induce and study deep vein thrombosis (DVT) in mice [3].

## 2. Material and Methods

### 2.1. Animals

All animal experiments were in accordance with the Declaration of Helsinki and National Institutes of Health guidelines. All experiments were approved by the Ethics Committee of the University Hospital Mainz and by the Institutional Animal Care and Use Committee (IACUC, Landesuntersuchungsamt Rheinland-Pfalz, Koblenz, Germany). All mice were housed in a barrier facility (Translational Animal Research Center (TARC), University Medical Center Mainz), kept in filter top cages with 2–5 mice per cage under specific pathogen-free (SPF) conditions.

C56BL/6 mice originally obtained from Jackson Laboratories (C57BL/6J; Bar Harbor, USA) were used as experimental animals. After treatment, animals were anesthetized by isoflurane inhalation (5% inhalant in room air) and killed by exsanguination via right ventricular puncture. The heart and lungs were rapidly excised, transferred to 4°C Krebs-HEPES-solution (pH 7.35 containing NaCl 99.01 mM, KCl 4.69 mM, CaCl_2_ 2.50 mM, MgSO_4_ 1.20 mM, NaHCO_3_ 25.0 mM, K_2_HPO_4_ 1.03 mM, Na-HEPES 20.0 mM, and D-glucose 11.1 mM), and cleared of adhesive tissue.

### 2.2. Isometric Tension Studies

To assess vasodilator properties of isolated pulmonary arterial segments, pulmonary arteries were cut into 3 mm segments and mounted on force transducers (Kent Scientific Corporation, Torrington, CT; PowerLab, ADInstruments, Spechbach, Germany) in organ chambers filled with Krebs–Henseleit solution (37°C, pH 7.35, containing 118 mM NaCl, 4.69 mM KCl, 1.87 mM CaCl_2_, 1.2 mM MgSO_4_, 1.03 mM K_2_HPO_4_, 25 mM NaHCO_3_, and 11.1 mM D-glucose) bubbled with carbogen gas (95% O_2_ and 5% CO_2_) and containing 1 *μ*M indomethacin to prevent endogenous synthesis of prostaglandins.

Following preconstriction with phenylephrine (0.15 *μ*M) or prostaglandin F2*α* (3 nM) to reach 50–80% of maximal tone induced by KCl, concentration-relaxation curves were recorded in response to increasing concentrations of the endothelium-dependent vasodilator acetylcholine (ACh, 1 nM–3 *μ*M).

### 2.3. Induction of DVT by Flow Restriction in the Inferior Vena Cava (IVC)

Male animals of at least 12 weeks of age with a minimum body weight of 25 g were anesthetized by intraperitoneal injection of a solution of midazolam (5 mg/kg; Ratiopharm GmbH, Ulm, Germany), medetomidine (0.5 mg/kg body; Pfizer Deutschland GmbH, Berlin, Germany), and fentanyl (0.05 mg/kg; Janssen-Cilag GmbH, Neuss, Germany). The animals were fixed on a custom-built stage and maintained at physiological temperature. For IVC flow restriction to induce DVT, animals were then depilated with hair removal cream at the area of surgery. A median laparotomy was performed, and the IVC was exposed by atraumatic surgery. We positioned a space holder (Asahi Fielder XT Guide Wire 0.014” [0.36 mm]; Abbot Vascular, Abbot Park, USA) on the outside of the vessel and placed a permanent narrowing ligature (7.0 monofil polypropylene filament, Prolene; Braun, Melsungen, Germany) exactly below the left renal vein. Subsequently, the wire was removed to avoid complete vessel occlusion. Side branches were left open in all groups [4]. To rule out endothelial injury as a trigger for venous thrombosis, all mice with bleedings or any injury of the IVC during surgery were excluded from further analysis. Median laparotomy was immediately sutured by a 7.0 polypropylene suture (Ethicon). After the surgical procedure, the animals were administered atipamezole (0.05 mg/kg) and flumazenil (0.01 mg/kg) intraperitoneally to antagonize anesthesia; postoperative analgesia was carried out with buprenorphine (0.075 mg/kg). For weight and length measurement of thrombi, animals were sacrificed 24 hours (h) after surgery, the IVC was excised below the renal veins, and the thrombus was exteriorized.

### 2.4. Induction of Pulmonary Embolism by Intravenous Thrombin Injection

Anesthesia of mice was induced in a chamber (2–4% isoflurane mixed with 0.2 L/min 100% O_2_) and maintained with a face mask (0.5–1.5% isoflurane with 0.05–0.1 L/min 100% O_2_). Animals were kept on a heated table mounted on a rail system (Visual Sonics, Toronto, Canada). Acute experimental pulmonary embolims were induced by retroorbital injection of 166 U/kg BW *α*-thrombin (bovine, Sigma-Aldrich, St. Louis, USA) in 100 *μ*L sterile saline. Immediately after each injection, echocardiography was performed as described below. Pulmonary embolisms after thrombin injection were confirmed by a sudden bradycardia or asystole, apnea or bradypnea, and an increase in PA pressure as observed by echocardiography. Animals were left to recover for 15 minutes before the next injection was performed. In total, up to three injections were conducted per animal. Our protocol was based on several factors: our aim was to induce a hemodynamically relevant, yet nonlethal, pulmonary embolism. In our hands, thrombin doses exceeding 166 U/kg BW were lethal in a significant number of animals, while after a single thrombin injection at or below this thrombin dose, PAP decreased rapidly towards normal levels (Figure S2). We thus aimed at delivering the pulmonary embolism in multiple, repetitive fractions. Three injections every 15 minutes emerged as the fastest protocol survived by the majority of animals.

### 2.5. Echocardiography and Vascular Sonography by High-Frequency Ultrasound (HFUS)

**Figure 1:**
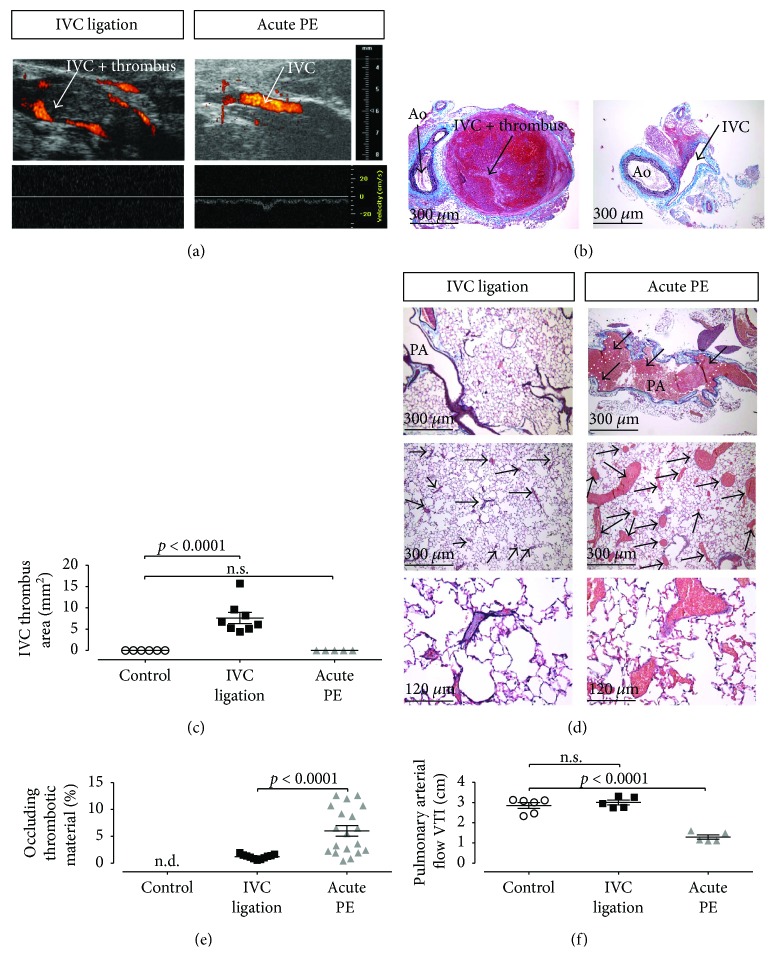
IVC ligation results in IVC thrombus formation with small pulmonary embolisms, whereas intravenous thrombin injection results in large pulmonary embolisms with significantly reduced PA flow. HFUS Power-Doppler imaging (a) and pulsed-wave Doppler imaging (a) or histologic analysis (b) of the IVC revealed a thrombus formation upstream of the subtotal IVC ligation with no detectable flow (left), while no flow reduction or thrombus in the IVC was detectable after induction of experimental pulmonary embolisms after intravenous thrombin injection ((a), (b) right subpanel, (c) quantification). (d) Histologic analysis of pulmonary tissue revealed numerous large thromboemboli after thrombin injection ((d) right subpanels) which involved the large pulmonary arteries, while small thromboemboli involving the peripheral pulmonary arteries could be observed after IVC ligation ((d) left subpanels). After thrombin injection, a significant obstruction of the pulmonary arterial vascular bed ((e) right) along with a significant reduction of the PA flow ((f) right) could be observed. After IVC ligation, the degree of pulmonary arterial obstruction was significantly less ((e) middle) than in PE mice and did not result in an impaired PA flow ((f) middle). PA: pulmonary artery; 5–8 animals per group. Data are presented as mean and SEM. 1-way ANOVA with Bonferroni's multiple comparison test.

Anesthesia of mice was induced in a chamber (2–4% isoflurane mixed with 0.2 L/min 100% O_2_) and maintained with a face mask (0.5–1.5% isoflurane with 0.05–0.1 L/min 100% O_2_). Animals were kept on a heated table mounted on a rail system (Visual Sonics, Toronto, Canada). Ultrasound was performed with the Vevo 770 System and a 40 MHz mouse scan head (RMV 706 for vascular sonography or RMV 707B for echocardiography; VisualSonics). Body temperature was monitored using a rectal probe and maintained at 37°C. The chest or the abdomen of the mouse was depilated, and warm ultrasound transmission gel was applied to enable visualization and optimize image quality. In the absence of a pulmonary valve stenosis or a right ventricular outflow tract obstruction, pulmonary arterial pressure (PAP) correlates to the right ventricular systolic pressure (RVSP). In mice, RVSP can be directly measured by invasive cannulation of the right ventricle via the jugular vein. This is a terminal procedure and therefore does not allow for the repetitive measurements required for our study. Estimation of PAP via the modified Bernoulli equation using the tricuspid valve regurgitation signal is not applicable, as mice do not exhibit a physiological tricuspid regurgitation like humans or larger animals. To overcome this, pulmonary arterial acceleration time (PAT) was assessed as a surrogate parameter for PAP/RVSP as described and verified by Thibault et al. [5]: two-dimensional images of the pulmonary infundibulum were obtained from the parasternal short-axis view at the level of the aortic, and pulsed-wave Doppler (pw-Doppler) recording of the pulmonary blood flow was obtained after positioning the pw-Doppler sample at the tip of the pulmonary valve leaflets and aligned to maximize laminar flow (Figure S1). As PAP/RVSP increases, PAT shortens. Importantly, estimation of PAP/RVSP is applicable in situations of acute and chronic increase of pulmonary pressure and is independent from heart rate or moderate anesthesia. PAT and RVSP where shown to exhibit a linear correlation (RVSP [mmHg] = −1.5 × PAT [ms] + 63.7). To facilitate understanding, results were presented as PAT (ms) and as calculated RVSP^∗^ (mmHg) with a cutoff of 21 ms (PAT)/32 mmHg (RVSP^∗^) between normal and elevated PAP/RVSP [5]. Apart from that, pulmonary arterial flow was displayed as the velocity-time integral (VTI [cm/s x s], see Figure 1).

For vascular sonography, first, a long-axis view was used to visualize the IVC, the ligation, and the formed thrombus. An optimal freeze-frame image was taken manually and, using the Vevo 770 software, the cross-sectional area of the clot was traced to obtain the measurement. The length, width, and area of clots were measured applying B-mode imaging.

### 2.6. RT-PCR

For isolation of RNA, snap-frozen mouse pulmonary arteries were homogenized with stainless steel micropesteles (A. Hartenstein, Würzburg, Germany), and the modified guanidine isothiocyanate method of Chomczynski and Sacchi [6] was used. RT-PCR was performed with the CFX96 Real-Time PCR Detection System (Bio-Rad, Munich, Germany). For RT-PCR analysis, 0.125 *μ*g of total RNA were used with the QuantiTect Probe RT-PCR kit (Qiagen, Hilden, Germany). TaqMan Gene Expression Assays were used as the probe and primer sets (Applied Biosystems, Foster City, CA) for beta actin (mouse: Mm00607939_s1), TATA-box binding protein (Tbp, mouse: Mm00446973_m-1), plasminogen activator inhibitor-1 (PAI-1, mouse: Mm00435858_m1), and gp91phox (Cybb, mouse: Mm01287743_m1). Results were quantified with the relative Ct method and normalized to the TATA-box binding protein.

### 2.7. Histochemistry and Immunohistochemistry

The lungs, hearts, and large vessels were fixed in situ by intratracheal instillation of paraformaldehyde (PFA) as described, embedded in paraffin, and sectioned. IVC thrombi were excised with the surrounding tissue and fixed, embedded, and serially sectioned at 5 *μ*m thickness. Slides were deparaffinized in xylene and rehydrated in serial ethanol concentrations. To visualize elastic fibers (black), fibrin or muscle (red), and fibrotic tissue (blue), a combined Verhoeff's Elastica- (VES-) Masson trichrome (MTC) stain was employed, as described [7]. Antigen retrieval was performed in citrate buffer (10 mmol/L sodium citrate, pH 6.0) for 20 min at 99°C. Slides were blocked with phosphate-buffered saline (PBS) with 0.05% Tween-20 and 1% bovine serum albumin (BSA). Primary antibody against Nox2/gp91^phox^ was purchased from BD Life Sciences (Clone 53, catalogue number 611415, Franklin Lakes, United States). Incubation with primary antibodies was conducted at 4°C overnight. Secondary antibodies were purchased from Abcam (ab150116). After three washes in PBS for 5 min each, slides were incubated with the secondary antibodies at room temperature in the dark for 1 hour. After three washes in PBS for 5 min each, the slides were counterstained with DAPI and mounted. The slides were allowed to dry for 24 h. Sections were imaged with an Olympus BX5 (VES-MTC) or an IX73 (all others) microscope.

### 2.8. Oxidative Fluorescent Microtopography

Isolated pulmonary trunks and arteries were cut into rings and incubated in Krebs-HEPES solution for 15 min at 37°C, embedded in aluminium cups of about 1 mL of a polymeric resin (Tissue-Tek O.C.T. compound, Sakura Finetek, Staufen, Germany), and frozen in liquid nitrogen. Cryosections (6 *μ*m) were stained with the superoxide-sensitive dye dihydroethidium (DHE, 1 *μ*M in PBS) and incubated for 30 min at 37°C. Green and red fluorescence was detected using a Zeiss Axiovert 40 CFL Camera (Zeiss, Oberkochen, Germany). Sections of all study arms were analyzed in parallel with identical imaging parameters.

### 2.9. Cell Culture Studies and In Vitro ROS Assays

Human pulmonary artery endothelial cells (HPAECs) were purchased from PromoCell (catalogue number C-12241; PromoCell, Heidelberg, Germany) and cultivated at 37°C under 5% CO_2_ in endothelial cell growth medium (PromoCell; catalogue number C-22010). ROS assays were performed as described [8]. Briefly, for DCF/DHE enhanced chemifluorescence, HPAECs were plated in clear bottom black 24-well plates (Corning, New York, USA). The following day, the medium was aspirated, and cells were serum-starved in physiological buffer in a humidified incubator at 37°C, 5% CO_2_ for 30 min.

For DCF enhanced fluorescence, the cells were switched to fresh medium containing 10 *μ*M DCF, for 10 min at 37°C in the dark. Finally, the DCF solution was removed, and cells were washed twice with pre-warmed buffer and stimulated with 0.5 units/mL thrombin at 37°C, 5% CO_2_ for 5 min. Cells were imaged immediately with an Olympus IX73 (excitation 485 nm and emission 525 nm). All the buffers contained 0.2 *μ*M L-NAME to prevent peroxynitrite formation, which is reported to interfere with DCF fluorescence [9].

For DHE fluorescence, cells were switched to pre-warmed buffer containing 5 *μ*M DHE and 0.5 U/mL thrombin at 37°C, 5% CO_2_ for 10 min. Cells were imaged immediately with an Olympus IX73 (excitation 520 nm and emission 590 nm). Cellular ROS levels were assessed by quantification of cellular fluorescence of 9–12 independent high-power fields (HPFs).

### 2.10. Statistics

Data are expressed as mean ± SEM. Statistical calculations were performed with GraphPad Prism 6 (GraphPad Software Inc., San Diego, CA). D'Agostino-and-Pearson normality test was first performed, and Pearson's correlation, Fisher's exact test, Mann–Whitney test, paired or unpaired *t*-test, Kruskal–Wallis test, 1-way ANOVA or 2-way ANOVA with post hoc Bonferroni's or Dunn's multiple comparison test were used as appropriate. Values of *p* < 0.05 were considered significant.

## 3. Results

### 3.1. Inferior Vena Cava Stenosis Results in DVT without Hemodynamically Relevant Pulmonary Embolism

We performed IVC ligation to induce stenosis of the vein with 80% flow reduction and reproducibly induced DVT in male C57BL/6 mice [4]. Importantly, operated mice developed only small thrombi in the pulmonary arterial bed as assessed by (VES-MTC) staining of the lung parenchyma 24 h post ligation. In contrast, single intravenous injection of *α*-thrombin (166 U/kg) into the retroorbital venous sinus reproducibly caused pulmonary embolisation without provoking DVT (Figures 1(a)–1(d)). This was mirrored by functional assessment of pulmonary arterial hemodynamics with a significant decrease of pulmonary artery (PA) flow in mice with acute PE (Figure 1(f)).

### 3.2. Intravenous Thrombin Injection Results in Pulmonary Embolism with Acute Pulmonary Hypertension

**Figure 2:**
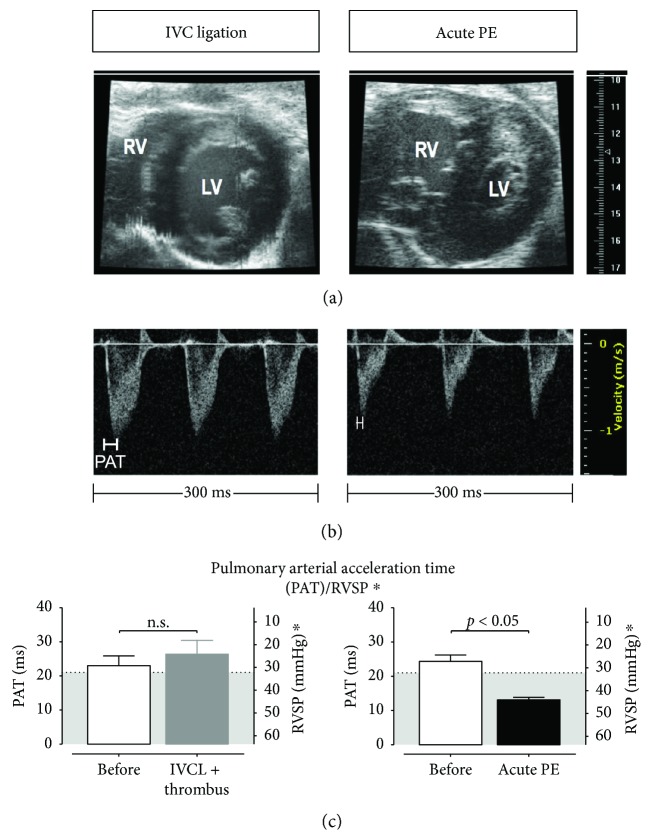
IVC ligation has no effect on pulmonary hemodynamics, while sublethal acute pulmonary embolism induced by intravenous thrombin injection results in pulmonary hypertension and right ventricular dysfunction. (a) left subpanel: B-mode imaging in parasternal short-axis view shows normal morphology of cardiac left and right ventricles (LV and RV, resp.), while noninvasive assessment of pulmonary arterial/right ventricular systolic pressure (RVSP) by measurement of pulmonary arterial acceleration time (PAT) did not reveal an increase in RVSP ((c) left subpanel). (b) left subpanel: representative PW-Doppler tracings 24 h after induction of IVC-thrombus formation by IVC ligation. Immediately after induction of sublethal acute PE by intravenous thrombin injection, cardiac imaging revealed RV enlargement ((a) right subpanel) correlating to a significantly decreased PAT indicating a significant increase in RVSP ((c) right subpanel, results are presented as PAT (ms) and as calculated RVSP^∗^ (mmHg), with a cutoff of 21 ms (PAT)/32 mmHg (RVSP^∗^) between normal and elevated PAP, with the grey area indicating the regions of elevated PAP [5]). (b) right subpanel: representative tracings. IVCL: IVC ligated mice. 5 animals per group; data are presented as mean and SEM. *t*-test.

Injection of thrombin not only caused PE but also led to hemodymic challenge of the right ventricle (RV), with dilatation of the RV, flattening of the intraventricular septum (so-called D-sign), and compression and compromise of the left ventricle (Figure 2(a)). Importantly, this was paralleled by a decrease in pulmonary arterial acceleration time (PAT) assessed by high-frequency ultrasound pulsed-wave Doppler imaging (Figures 2(b) and 2(c)), which is inversely proportional to an increase in PAP. In contrast, DVT caused by ICV ligation did not induce either hemodynamic challenge to the RV or an increase in PAP (Figures 2(a)–2(c)).

### 3.3. Repetitive PEs Induce Sustained Pulmonary Hypertension and Endothelial Dysfunction of the Pulmonary Arteries

**Figure 3:**
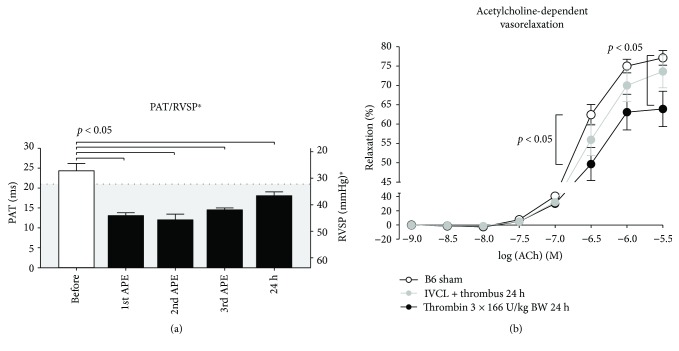
Repeated thrombin injection led to recurrent PE and sustained PA pressure elevation and endothelial dysfunction. While after a single thrombin injection, symptoms were generally resolved and PA pressure normalized within minutes, the time to recovery lengthened with every injection. 24 h after (triple) pulmonary embolisms, PAT remained significantly shortened, indicating a persisting elevation of pulmonary pressure ((a) are presented as PAT (ms) and as calculated RVSP^∗^ (mmHg), with a cutoff of 21 ms (PAT)/32 mmHg (RVSP^∗^) between normal and elevated PAP, with the grey area indicating the regions of elevated PAP [5]). 5 animals per group. Data are presented as mean and SEM. 1-way ANOVA and Bonferroni's multiple comparison test. (b) Isometric tension studies revealed a significant endothelial dysfunction as compared to untreated mice in PAs explanted from embolised animals. APE: acute PE; PAT: pulmonary arterial acceleration time. 5 animals per group. Data are presented as mean and SEM. 2-way ANOVA and Dunn's multiple comparison test.

Next, we investigated the feasibility of our approach for induction of sustained pulmonary arterial hypertension (PAH). We repeatedly injected *α*-thrombin (166 U/kg BW) at timepoint 0, +15 min, and +30 min and assessed PAT by high-frequency ultrasound immediately after each injection and 24 h later. We established sustained PAH at 24 h with significantly shortened PAT in mice with repeated PEs compared to baseline (Figure 3(a)). This PAH caused by recurrent PE was paralleled by increased pulmonary arterial dysfunction. Isometric tension studies of isolated segments of the pulmonary artery of mice with repeated PE revealed desensitization to the endothelium-dependent vasodilator acetylcholine (Figure 3(b)) compared to control mice. This pulmonary artery endothelial dysfunction 24 h after the intervention was also detectable in IVC ligated mice with DVT by trend, although it did not significantly differ from controls.

### 3.4. Increased Vascular Superoxide Formation in Pulmonary Arteries after Repeated Pulmonary Embolism

**Figure 4:**
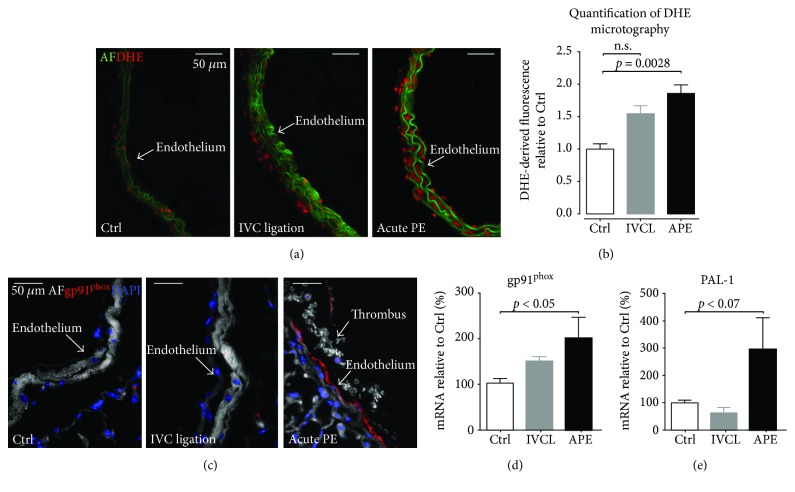
Pulmonary arterial oxidative stress is mediated by gp91^phox^ NADPH oxidase. (a and b) Microtopography revealed significantly increased superoxide levels in PAs from triple-embolised mice as compared to PAs from control mice or after IVC ligation. IHC (c) and qPCR (d) revealed a significantly increased NOX2/gp91^phox^ expression in PAs after pulmonary embolism as compared to PAs from control mice or after IVC ligation. (e) qPCR of PAI-1 mRNA in PAs. Ctrl: control mice; AF: autofluorescence; DHE: dihydroethidium (a); gp91^phox^: antibody against gp91^phox^ NADPH oxidase; DAPI: 4′,6-diamidin-2-phenylindol; IVCL: IVC ligated mice; APE: mice with recurrent acute PE. 5 animals per group; data are presented as mean and SEM. 1-way ANOVA and Bonferroni's multiple comparison test.

**Figure 5:**
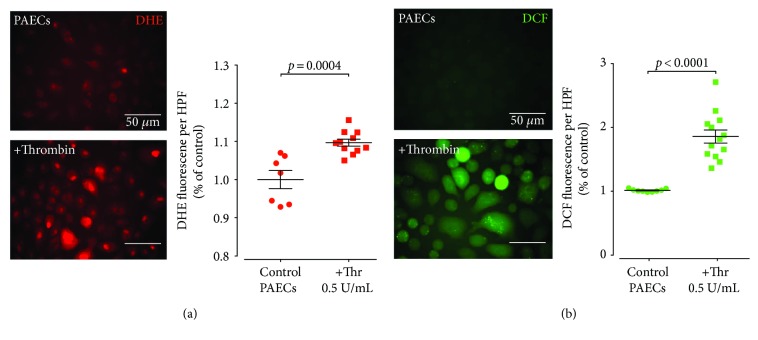
Thrombin induces increased levels of reactive oxygen species (ROS) in pulmonary arterial endothelial cells (PAECs). Exposure of cultured human PAECs to thrombin resulted in increased levels of ROS as determined by the fluorescent probes DCF and DHE ((a, b) left representative images, (a, b) right quantification). Thr: thrombin. 9–12 high-power field acquisitions per group. Data are presented as mean and SEM. Mann–Whitney test.

It is well established that endothelial dysfunction can be caused by a dysequilibrium of increased superoxide formation and challenged scavenger systems, which results in loss of nitric oxide bioavailability. We therefore assessed vascular superoxide formation with dihydroxyethidium staining and expression of the most important vascular source of superoxide, the gp91^phox^ NADPH oxidase. Repeated PEs induced a significant increase in pulmonary artery superoxide formation (Figures 4(a) and 4(b)), whereas this change was only moderate in IVC ligated mice. Immunohistochemistry of pulmonary artery segments revealed an increased expression of gp91^phox^ in mice with recurrent PE but not in mice with IVC ligation (Figure 4(c)). This finding was confirmed by an increased mRNA expression of the gp91^phox^ in pulmonary artery homogenates of recurrent PE mice but not IVC ligated mice (Figure 4(d)). Thrombin is known to induce plasminogen activator inhibitor-1 (PAI-1), a modulator and surrogate marker of vascular fibrosis and—along with thrombin—an inducer of gp91^phox^ expression. Conclusively, PAI-1 mRNA expression in pulmonary artery homogenates was significantly increased in recurrent PE mice but not in IVC-ligated mice, compared to controls. (Figure 4(e)). Elevated levels of superoxide production were also confirmed in human pulmonary artery endothelial cells in response to thrombin stimulation (Figure 5).

## 4. Discussion

The pathogenesis of CTEPH remains elusive; however, vascular dysfunction and remodelling as a sequel of repeated PE has been discussed as the main driver of the disease. It is well established that endothelial dysfunction (defined as a loss of nitric oxide bioavailability due to impaired biosynthesis and/or increased breakdown by superoxide radicals) is an early hallmark of systemic arterial disease in atherosclerosis, diabetes mellitus, or arterial hypertension [10]. It is widely accepted that a dysfunctional endothelium is a prerequisite for atherogenesis by allowing adhesion and transmigration of leukocytes into the vasculature [11] and that assessment of endothelial dysfunction in patients with cardiovascular disease is useful for risk assessment and secondary prevention [12, 13].

In PAH, the potential role of endothelial dysfunction is less well understood. In hypoxia-induced pulmonary arterial hypertension, the remodelling of the pulmonary arteries has been ascribed to increased endothelial-to-mesenchymal transition of the pulmonary arterial endothelial cells in mice and humans [14]. This has been associated with a disturbed barrier function of PA endothelial cells and an increase in proinflammatory cytokines, like tumor necrosis factor *α* and interleukin 6 [14], which are known to increase NADPH oxidase expression and activity and reduce NO bioavailability. Interestingly, the functional sequel has been tested in isolated segments of pulmonary arteries obtained from humans with a history of smoking and chronic obstructive pulmonary disease. Compared to controls, acetylcholine-dependent vasorelaxation was drastically impaired in the PA segments of the affected patients [15]. Isolated rings of PAs obtained from rats exposed to intermittent hypoxia were characterized by endothelial dysfunction with desensitization to acetylcholine in isometric tension studies [16]. However, a clear interconnection between DVT or pulmonary embolisation, pulmonary hypertension, and PA endothelial dysfunction has never been demonstrated. To the best of our knowledge, our study is the first description of DVT- or PE-induced endothelial dysfunction in pulmonary arteries in mice assessed by isometric tension studies of isolated PA segments.

DVT is the most common cause of pulmonary embolism, and ligation of the IVC to induce stenosis of the vein is currently regarded as the best mouse model to reflect human pathophysiology of DVT [4, 17–19]. Therefore, studying the IVC ligation model appears both obvious and very suitable to set up an experimental model of pulmonary embolism. Interestingly, in our study, robust induction of DVT in the IVC resulted in numerous, but only small, embolisations in the peripheral pulmonary arteries. It did not result in hemodynamically relevant pulmonary embolisms leading to PAH and only caused very mild pulmonary arterial endothelial dysfunction. This corroborates the existing evidence in literature, which has repeatedly reported on the hardships to induce PE in mice and other animals [20]. This phenomenon has best been described in dogs, which are resistant to sustained DVT or PE because of high endogenous thrombolytic activity [21], probably caused by increased plasmin and/or plasminogen activity [22]. A repeated combination of localized transcatheter thrombin application in deep veins, mechanical vein obstruction, and systemic plasmin antagonization by tranexamic acid was able to induce sustained PEs in dogs and was termed chronic pulmonary thromboembolism by the authors. However, in contrast to CTEPH, the relative increase in PAH was only modest in the affected dogs, and it is a very cumbersome method that comes at a high mortality rate with many protocol deviations [23]. Based on these experiences, intravenous thrombin injection (10 U/25 g body weight) combined with perfluorocarbon nanoemulsions that was detectable by magnetic resonance imaging was used to induce PE in mice [24]. However, whether this procedure also induced PAH or PA endothelial dysfunction remained unclear.

We have used a similar approach to induce PE, repeatedly injecting *α*-thrombin at a dose of 166U/kgBW intravenously via the retroorbital venous sinus. This procedure caused both PAH and PE and was associated with a significant increase in NADPH oxidase expression and superoxide formation in the PA, suggesting gp91^phox^ gene induction by thrombin itself [25], fibrin, or other cellular or plasmatic components of the clot. Interestingly, DVT in IVC ligated mice also caused mild endothelial dysfunction in the PA combined with increased superoxide formation and gp91^phox^ activity. However, as the small thromboemboli observed in this model were located distally of the pulmonary arteries, this implies that systemic traces of thrombin, fibrin, or fibrin breakdown products reach the pulmonary circulation to induce PA endothelial dysfunction, potentially via a PAI-1-dependent mechanism. PAI-1 can be induced by thrombin [26]; it is a driver of vascular remodelling [27] and may also act as an inducer of gp91^phox^ gene expression via protease activated receptor signaling [28]. Importantly, this concept was further supported by our translational findings utilizing a human cell culture model: already short-term exposure to thrombin resulted in significantly increased ROS production in cultured PAECs (Figure 5).

We conclude that oxidative stress mediated by NADPH oxidase-produced superoxide anions and endothelial dysfunction of the pulmonary arteries are closely linked to PAH and occur early after pulmonary embolism. It even occurs in DVT in the absence of hemodynamically relevant PE, indicating a very early sign of vascular remodelling in pulmonary circulation in remote venous clotting. In summary, our study is the first to provide a link between pulmonary embolism, pulmonary arterial hypertension, and pulmonary arterial endothelial dysfunction and oxidative stress in mice. We established a new mouse model to induce PE that could help to develop a model of CTEPH in mice.

## Supplementary Materials

Supplementary Materials Figure S1: Noninvasive assessment of PAP. To allow for repetitive measurements of PAP, pulmonary arterial acceleration time (PAT) was assessed as a surrogate parameter for PAP/RVSP as described and verified by Thibault et al. [5]: two-dimensional images of the pulmonary infundibulum were obtained from the parasternal short-axis view at the level of the aortic, and pulsed-wave Doppler (pw-Doppler) recording of the pulmonary blood flow was obtained after positioning the pw-Doppler sample at the tip of the pulmonary valve leaflets and aligned to maximize laminar flow (Figure S1). As PAP/RVSP increases, PAT shortens. PAT and RVSP where shown to exhibit a linear correlation (RVSP [mmHg] = −1.5 × PAT [ms] + 63.7). To facilitate understanding, results were presented as PAT (ms) and as calculated RVSP^∗^ (mmHg) with a cutoff of 21 ms (PAT)/32 mmHg (RVSP^∗^) between normal and elevated PAT [5]. Figure S2: short-term changes of pulmonary pressure after repetitive sublethal pulmonary embolisms. Continuous measurements of PAT revealed a sudden PAP increase upon intravenous injection of sublethal thrombin doses (166 U/kg BW). Importantly, after a single thrombin dose, PAP decreased rapidly towards normal levels, while after a second injection, PAP-normalization was decelerated. Results are presented as PAT (ms) and as calculated RVSP^∗^ (mmHg) with a cutoff of 21 ms (PAT)/32 mmHg (RVSP^∗^) between normal and elevated PAT [5].
