# Supplementary material for: Pulmonary Arterial Hypertension and Endothelial Dysfunction Is Linked to NADPH Oxidase-Derived Superoxide Formation in Venous Thrombosis and Pulmonary Embolism in Mice
Source: Oxid Med Cell Longev. 2018 Jun 10;2018:1860513. doi: 10.1155/2018/1860513 (PMC6015670; doi:10.1155/2018/1860513)
Supplement: Supplementary Materials — Figure S1: Noninvasive assessment of PAP. To allow for repetitive measurements of PAP, pulmonary arterial acceleration time (PAT) was assessed as a surrogate parameter for PAP/RVSP as described and verified by Thibault et al. [5]: two-dimensional images of the pulmonary infundibulum were obtained from the parasternal short-axis view at the level of the aortic, and pulsed-wave Doppler (pw-Doppler) recording of the pulmonary blood flow was obtained after positioning the pw-Doppler sample at the tip of the pulmonary valve leaflets and aligned to maximize laminar flow (Figure S1). As PAP/RVSP increases, PAT shortens. PAT and RVSP where shown to exhibit a linear correlation (RVSP [mmHg] = −1.5 × PAT [ms] + 63.7). To facilitate understanding, results were presented as PAT (ms) and as calculated RVSP∗ (mmHg) with a cutoff of 21 ms (PAT)/32 mmHg (RVSP∗) between normal and elevated PAT [5]. Figure S2: short-term changes of pulmonary pressure after repetitive sublethal pulmonary embolisms. Continuous measurements of PAT revealed a sudden PAP increase upon intravenous injection of sublethal thrombin doses (166 U/kg BW). Importantly, after a single thrombin dose, PAP decreased rapidly towards normal levels, while after a second injection, PAP-normalization was decelerated. Results are presented as PAT (ms) and as calculated RVSP∗ (mmHg) with a cutoff of 21 ms (PAT)/32 mmHg (RVSP∗) between normal and elevated PAT [5]. [file 1860513.f1.pdf]

Supplementary figures:

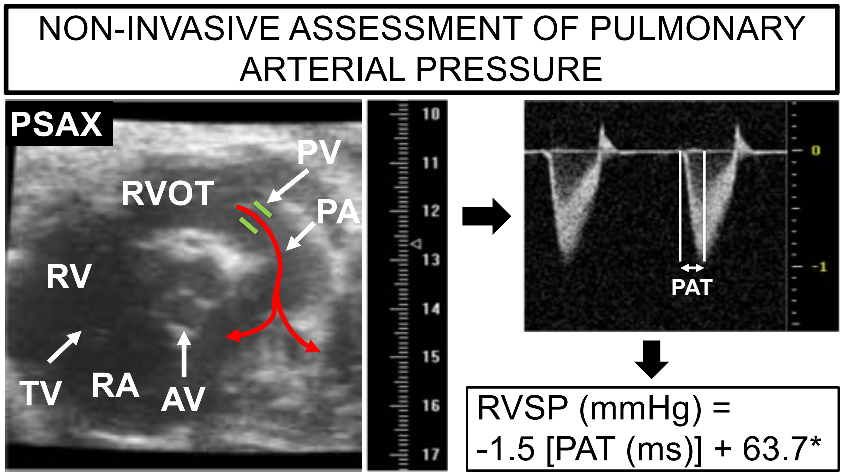

**PSAX:**	**Parasternal short axis view**	**PA:**	**pulmonary artery**
**AV:**	**Aortic valve**	**→:**	**Direction of blood flow**
**RA:**	**Right atrium**	**=:**	**Position of pw-Doppler probe**
**TV:**	**Tricuspid valve**	**PAT:**	**Pulmonary arterial acceleration time**
**RV:**	**Right ventricle**		
**RVOT:**	**RV outflow tract**	**RSVP:**	**Right ventricular systolic pressure**
**PV:**	**Pulmonary valve**		

Figure S1: Non-invasive assessment of PAP. To allow for repetitive measurements of PAP, pulmonary arterial acceleration time (PAT) was assessed as a surrogate parameter for PAP/RVSP as described and verified by Thibault et al. [5]: Two-dimensional images of the pulmonary infundibulum were obtained from the parasternal short-axis view at the level of the aortic and pulsed-wave Doppler (pw-Doppler) recording of the pulmonary blood flow was obtained after positioning the pw- Doppler sample at the tip of the pulmonary valve leaflets and aligned to maximize laminar flow (Figure S1). As PAP/RVSP increases, PAT shortens. PAT and RVSP where shown to exhibit a linear correlation (RVSP [mmHg] = -1.5 x PAT [ms] + 63.7). To facilitate understanding, results were presented as PAT (ms) and as calculated RVSP* (mmHg) with a cutoff of 21 ms (PAT)/ 32 mmHg (RVSP*) between normal and elevated PAT [5].

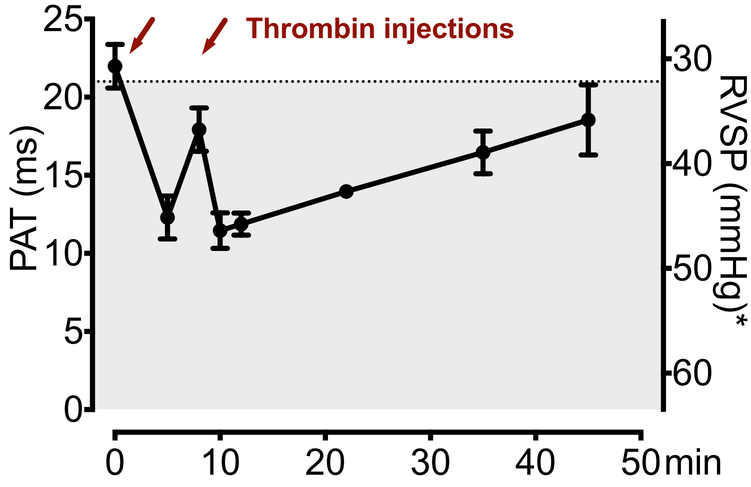

Figure S2: Short term changes of pulmonary pressure after repetitive sublethal pulmonary embolisms. Continuous measurements of PAT revealed a sudden PAP increase upon intravenous injection of sublethal thrombin doses (166 U/kg BW). Importantly, after a single thrombin dose, PAP decreased rapidly towards normal levels while after a second injection, PAP-normalization was decelerated. Results are presented as PAT (ms) and as calculated RVSP* (mmHg) with a cutoff of 21 ms (PAT)/ 32 mmHg (RVSP*) between normal and elevated PAT [5].
